# Reply to Paulo Schiavom Duarte

**DOI:** 10.1007/s00259-021-05469-y

**Published:** 2021-06-22

**Authors:** Julian M. M. Rogasch, Carsten Kobe

**Affiliations:** 1 Department of Nuclear Medicine, Charité – Universitätsmedizin Berlin, Corporate Member of Freie Universität Berlin, Humboldt-Universität Zu Berlin, and Berlin Institute of Health, Augustenburger Platz 1, D-13353 Berlin, Germany; 2 Berlin Institute of Health (BIH), Berlin, Germany; 3 Department of Nuclear Medicine, University Hospital of Cologne, Cologne, Germany

Dear Sir,

We thank Paulo Duarte for his interest and appreciate his comments regarding our review “Moving the goalposts while scoring―the dilemma posed by new PET technologies.”

The aim of our review was to highlight the clinical impact of new technologies for positron emission tomography (PET). We agree with Paulo Duarte that not every clinical aspect of a diagnostic tool such as PET or of new technologies developed for this modality require the same degree of clinical evidence as that outlined by Fryback and Thornbury [1] and demanded for therapeutic drugs. However, when it comes to technical innovations in the field of PET as an existing imaging modality, we as a nuclear medicine community should not be content simply with possible improvements in certain aspects of image quality or lesion detectability. If there are considerable benefits associated with new PET technologies, they should ultimately be of demonstrable value for patient care and patient-relevant outcomes.

We also agree with our colleague that the definition of benefit for the patient may vary between different clinical settings. In this regard, a benefit would not be restricted to prognosis (survival) but might be manifested in an earlier diagnosis of relapse (if this is advantageous to the treatment), higher lesion detection rates (if this results in relevant treatment changes), or even in a reduction of the administered activity and radiation exposure.

Paulo Duarte further suggests that diagnosis and prognosis of a disease have a value of their own. We have convincing data showing that the use of PET is beneficial for patients and can improve patient survival in several settings. In contrast, there is a lack of evidence for further improvement with “new PET technologies.” The mere observation that new PET technologies might have clinical consequences suggests that they should not be used without careful thought. Although this may appear overly cautious, such caution is warranted as it could prevent potential disadvantageous effects, and it is especially important in clinical scenarios in which treatment decisions are influenced by variations in image characteristics. The potentially higher lesion detection rates associated with the new PET technologies are also a concern. In this regard, one prominent and painful example of clinical problems resulting from the transition to new technologies was with PET in lymphoma, where the original introduction of PET had allowed us to omit radiotherapy in many patients, but the percentage of patients undergoing radiotherapy rebounded on stepwise introduction of emerging PET technologies.

This is why we support all efforts to ensure imaging comparability, as put forward by the EARL initiative, and we recommend being adequately cautious and continuously aware of the possible clinical impact of new PET technologies.

Best regards,

Julian Rogasch and Carsten Kobe.
